# RNA Recognition and Stress Granule Formation by TIA Proteins

**DOI:** 10.3390/ijms151223377

**Published:** 2014-12-16

**Authors:** Saboora Waris, Matthew Charles James Wilce, Jacqueline Anne Wilce

**Affiliations:** Department of Biochemistry and Molecular Biology, Monash University, Victoria 3800, Australia; E-Mails: saboora.waris@monash.edu (S.W.); matthew.wilce@monash.edu (M.C.J.W.)

**Keywords:** RNA-binding proteins, mRNA, stress granule, translation, 5'-oligopyrimidine terminal 5'-oligopyrimidine (TOP) elements, RNA-recognition motif (RRM), T cell restricted intracellular antigen-1 (TIA-1), TIA-1 related protein (TIAR), prion-related domain (PRD)

## Abstract

Stress granule (SG) formation is a primary mechanism through which gene expression is rapidly modulated when the eukaryotic cell undergoes cellular stresses (including heat, oxidative, viral infection, starvation). In particular, the sequestration of specifically targeted translationally stalled mRNAs into SGs limits the expression of a subset of genes, but allows the expression of heatshock proteins that have a protective effect in the cell. The importance of SGs is seen in several disease states in which SG function is disrupted. Fundamental to SG formation are the T cell restricted intracellular antigen (TIA) proteins (TIA-1 and TIA-1 related protein (TIAR)), that both directly bind to target RNA and self-associate to seed the formation of SGs. Here a summary is provided of the current understanding of the way in which TIA proteins target specific mRNA, and how TIA self-association is triggered under conditions of cellular stress.

## 1. Introduction

Eukaryotic cells are able to respond rapidly to environmental stresses at the level of mRNA translation. Heat, oxidative stress, viral infection, starvation or accumulation of unfolded proteins can all result in the stalling of translation followed by the swift sequestration of mRNA transcripts and their associated proteins into cytoplasmic foci known as stress granules (SG) [1,2]. Here, no translation takes place but the mRNA is protected from degradation. This allows the cells to direct their energy into translating mRNA that encode proteins that refold denatured proteins or repair oxidative damage. In particular, mRNA encoding heat shock proteins and some transcription factors are specifically excluded from SGs. When the stress passes, the SGs are disassembled, releasing the mRNA and allowing translation to resume. In this way cells can survive the adverse event.

There are also other specialised functions of SGs reported. In T cells, cytokine mRNA is sequestered into SGs to prime the cell for rapid cytokine translation and secretion upon T cell receptor stimulation [3]. In addition to sequestering mRNA, SGs also exert effects through the sequestration of signalling proteins. An anti-inflammatory response associated with SG formation is reported to occur not so much due to translational inhibition, but to the recruitment of TNF receptor-associated factor 2 (TRAF2) into SGs resulting in the blockage of nuclear factor kappa beta (NF-κB) signal transduction [4]. Similarly, when the protein receptor for activated C kinase 1 (RACK1) is sequestered into SG, MAPK kinase kinase (MTK1) apoptotic signalling, that depends upon RACK1 interactions, is reduced [5].

The importance of SG to the health of the cell is seen in disease states where SG function is compromised. SGs are the target of several RNA viruses that disrupt SGs to facilitate their own replication. In many cases this appears to serve the purpose of suppressing the host cell stress response against the virus [6]. SG formation that occurs in some cancers potentially assists cell survival by the sequestration of apoptotic factors [7,8]. SG formation is also linked to neurodegenerative disease, where associations between SG and intracellular protein aggregates are observed to occur [9,10]. Thus, the mechanism of SG formation and its proper regulation are important aspects of cellular biology for understanding disease progression.

SGs are similar to other types of mRNP granules (including processing bodies, germ granules and neuronal transport granules but are distinct in their composition [11,12]. SGs are characterized by the presence of key translational initiation factors including eIF4E, eIF4G, eIF4A, eIF4B, eIF3, eIF2, poly(A)-binding protein (PABP) and stalled 40S ribosomal units [6,13]. A distinguishing feature is the prominence of O-linked *N*-acetylglucosamine modification of many SG proteins [14]. Furthermore, SG comprise a host of RNA binding proteins including, Human antigen R (HuR), Tristetraprolin (TTP), Y box binding protein 1 (YB1), fragile X mental retardation protein (FMRP), AU-binding factor 1 (AUF1) and K-homology splicing regulator protein (KSRP) [15,16]. Many of these are passenger proteins that may not function directly in SG formation. SGs also consist of SG-nucleating proteins such as Ras GTP ase-activating protein-binding protein 1 (G3BP1), T cell restricted intracellular antigen-1 (TIA-1), TIA-1 related protein (TIAR) and Tudor domain-containing protein 3 (TDRD3) [17,18,19,20].

The best known proteins integral to SG formation are the TIA proteins that are considered robust markers of SGs [21,22]. TIA-1 and TIAR have the capacity to both bind to specific targeted mRNA transcripts and to nucleate SG formation via their prion-related *C*-terminal domain. Other functions of TIA proteins include translational repression of inflammatory mediators and the direction of splicing of targeted mRNA transcripts [23,24]. This review, however, focuses on the role of TIA protein in SGs with particular attention paid to our current understanding of the molecular basis of RNA recognition and SG formation by TIA proteins as determined through biophysical experimentation.

## 2. The T Cell Restricted Intracellular Antigen (TIA) Proteins

TIA proteins are approximately 375 amino acid ubiquitous RNA-binding proteins, predominantly expressed in brain, testis and spleen [25]. They possess three *N*-terminal RNA-recognition motifs (RRMs) and a *C*-terminal glutamine-rich prion-related domain (PRD) and share greater than 80% sequence homology overall. The RRMs (1–3) of TIA-1 and TIAR are 79%, 89% and 91% identical, while PRDs share 51% identity. The TIA proteins exert oligonucleotide binding via these RRMs and the PRD is essential for SG formation through self-association of this Q-rich domain [18]. TIA proteins lacking the PRD do not initiate SG formation, and substitution of the PRD for another prion-like domain reconstitutes the ability for SG formation. It is thus established that SG assembly can be mediated by prion-like interactions of the PRD of TIA proteins.

**Figure 1:**
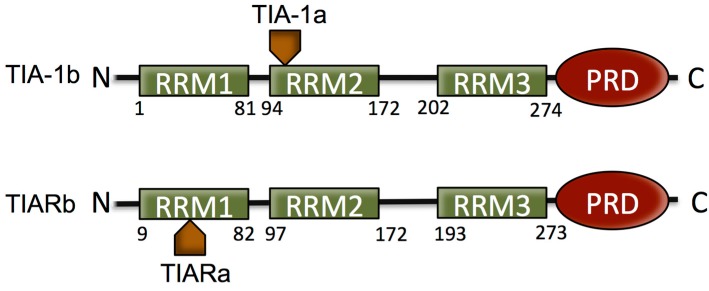
Schematic of the domain structure of T cell restricted intracellular antigen (TIA) protein isoforms. TIA proteins consist of three RNA recognition motifs (RRMs) that provide RNA/DNA binding specificity and a Q-rich prion-related domain (PRD) domain, involved in stress granule (SG) formation. The two isoforms of both proteins that form as a result of alternative splicing are indicated.

Both TIA-1 and TIAR occur with two isoforms formed as a result of alternative splicing. The inclusion of 11 amino acid residues at the beginning of RRM2 of TIA-1 is termed isoform TIA-1a, and the isoform without the 11 amino acids is termed isoform TIA-1b. TIAR containing 17 amino acids within RRM1 is referred to as isoform TIARa, and that lacking these 17 amino acids is isoform TIARb [25]. It has been determined that the relative expression of the two isoforms of TIA proteins varies in different tissues and cells and that the splicing activity differs between the difference TIA-1 isoforms [26] (Figure 1).

## 3. Stress Granule Formation in the Cell

**Figure 2:**
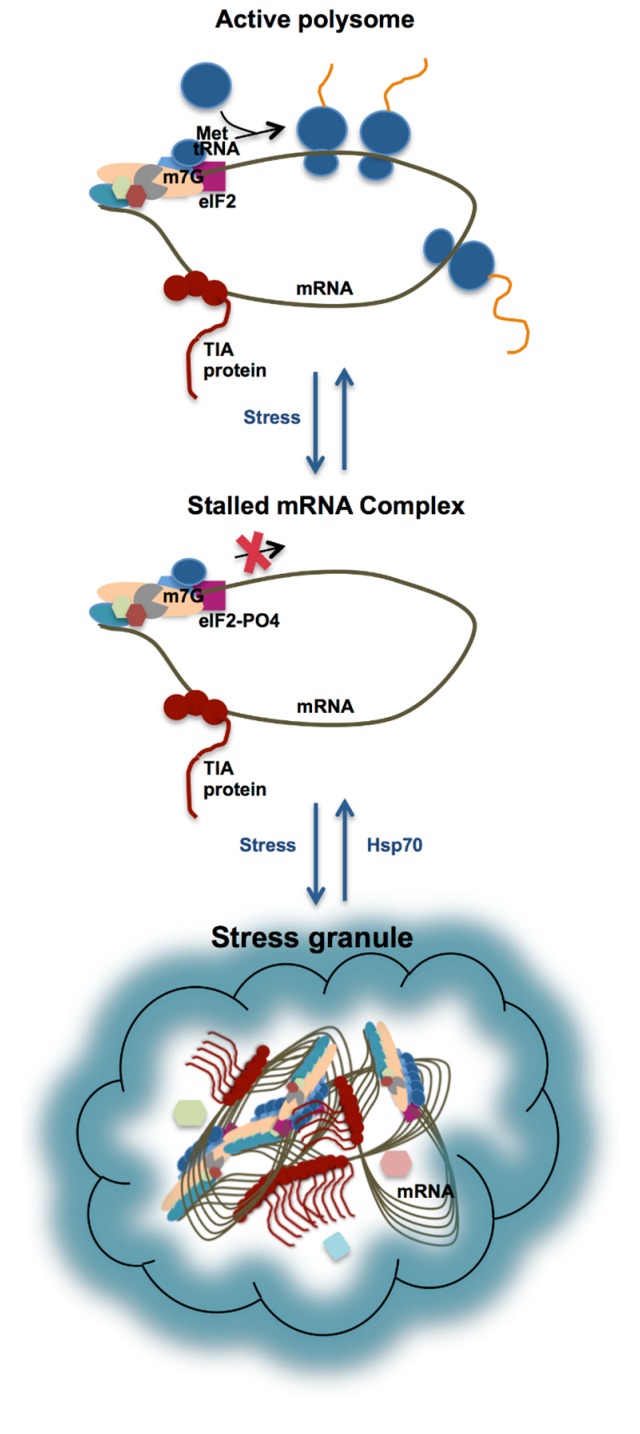
Schematic of SG formation formed via the self-association of TIA proteins (shown in brown with circles representing RRM domains, and a tail representing the *C*-terminal PRD) bound to stalled ribonucleoprotein-mRNA complexes. Upon phosphorylation of eIF2 under conditions of stress, the pre-initiation complex is stalled. Accumulation of the stalled mRNA complex results in its incorporation into SG (involving interactions between the TIA protein PRDs) until such time that heat shock proteins are able to reverse the process and translation is resumed.

The sequence of events that leads to SG formation is characterized to some extent. TIA proteins are components of regulatory complexes that specifically bind to adenine/uridine rich elements (AREs) present in 3'-untranslated regions of RNA [27]. TIA proteins shuttle continuously between the nucleus and cytoplasm, but upon stress accumulate in the cytoplasm. At the same time, stress causes ribosomes to be stalled. The best established mechanism for this is through the activation of kinases that phosphorylate the eukaryotic initiation factor 2 (eIF2) preventing the initiation of mRNA translation [11,28]. This results in the accumulation of stalled 48S pre-initiation complexes that include mRNA and their associated mRNA binding proteins [29] (Figure 2). These are then sequestered into SG through the self-association of the PRD. More recently, TIA-1 and TIAR have also been found to themselves arrest translation at the initiation step, through the binding of terminal 5'-oligopyrimidine (TOP) elements under periods of stress [30]. TOP elements occur predominantly in mRNA encoding ribosomal proteins and translation factors that are not required during stress.

SGs only persist in the cell for a limited time. After SGs have formed, the cell engages in the production of heat shock/chaperone proteins (Hsp70). These have the effect of protecting proteins from denaturation, and also begin to reverse the self-association of the TIA proteins, disassembling the SG after several hours once the stress is alleviated, allowing translation of the mRNA transcripts to resume [11,31].

Despite this understanding of the role of TIA proteins in SG assembly, there are important aspects of the molecular mechanism of this process that are not understood. The first relates to the basis of recognition of specific mRNA transcripts by the TIA proteins for their selective sequestration into SG. The second relates to the detailed molecular mechanism of self-association by the PRD. In some way, presumably, these two functions are integrated. A better understanding of mRNA recognition, self-association and disassembly of TIA proteins would help to understand the regulation of SG formation potentially leading to ways in which SG may be modulated in disease.

## 4. Specificity of TIA Proteins for Target mRNA

The subsets of mRNA that are sequestered into SG by TIA proteins are defined by the presence of specific motifs present in their 3'- or 5-untranslated regions (UTRs). TIA proteins were originally identified as having specificity for U-rich sequences based o *in vitro* selection and filter binding assays [32]. Such AU-rich RNA regulatory elements are commonly found in the 3'- and 5'-UTRs of mRNAs subject to rapid changes in stability and translation, including those that encode cytokines, pro-inflammatory mediators, stress-response proteins, extracellular matrix remodelling enzymes, and oncoproteins [33,34,35,36]. Indeed, upon the depletion of TIA proteins in cells there is massive up-regulation of cytokines, chemokines and growth stimulating factors [37]. Analysis of *in vivo* targets of TIA-1 (via immunoprecipitation and microarray identification of TIAR-bound transcripts) also verified the association of TIA proteins with U-rich transcripts [27]. In the case of TIAR, a C-rich motif was also discovered to be a target. TIAR was found to be associated with C-rich containing mRNA transcripts in unstressed cells, but upon UV stress these transcripts were released [38]. In studies of West Nile virus RNA binding by TIA proteins short AU sequences were identified [39]. Biophysical work conducted using surface plasmon resonance (SPR) verified the binding of TIAR to both AU-rich, U-rich and C-rich RNA, providing detailed insight into the relative affinity for the different target sequences and the kinetics of interaction [38,40]. Furthermore, the finding that TIA-1 and TIAR bind to terminal 5'-oligopyrimidine (TOP) elements under periods of stress, is consistent with the ability of TIA proteins to bind to C-rich and well as AU-rich elements [30].

The contribution of each of the three RRM domains to mRNA target binding has also been the subject of investigation. Notably, each single TIA protein RRM does not contribute equally to oligo-nucleotide binding. Dember *et al.*, showed that the three RRMs of TIA proteins contribute variously to the interaction with U-rich RNA [32]. They established that RRM2 is sufficient and necessary for binding to U-rich RNA. This is in agreement with work by Kim *et al.*, assessing TIAR binding to U-rich and AU-rich sequences [38,40] and that of Bauer *et al.*, who showed variable involvement of TIA-1 RRM domains [41]. It has been shown that U-rich RNA binding to a TIAR construct with all three domains (TIAR123) occurs with equivalent affinity to binding by TIAR lacking the third domain (TIAR12) demonstrating there is little or no contribution to binding by RRM3 to U-rich domains. Likewise, a TIAR construct comprising domains 1 and 2 (RRM12) binds to U-rich RNA with only marginally higher affinity than RRM2 alone, showing that RRM1 contributes little to U-rich RNA binding [42].

From this the roles of RRM1 and RRM3 are unexplained. However, further investigation revealed that RRM1 is, in fact, able to bind to T-rich ssDNA. Suswam *et al.*, showed that TIA-1 RRM1 is likely to play a role in the nucleus of the cell, binding to T-rich ssDNA and bringing TIA-1into close proximity to U-rich RNA as it is transcribed [43]. This possibly relates to its function in splicing and also suggests that the loading of TIA proteins to target RNA occurs very early in the biogenesis of mRNA. A further study of TIAR RRM1 interactions with DNA confirms that binding occurs with micromolar affinity [42]. Thus, this is an unusual case of an RRM preferentially binding DNA over RNA.

In the case of RRM2, it has been shown that binding occurs preferentially to both U-rich RNA and T-rich DNA, but that the affinity depends upon the presence of amino acids beyond the structured region of the RRM domain [42,44]. NMR titration experiments have identified the amino acid residues within the *C*-terminal extension of TIAR RRM2 that are perturbed by RNA binding and SPR has been used to demonstrate a 25-fold increase in affinity of the complex when these residues are present [42]. The *C*-terminal region is thought to provide extra contacts to the bound oligonucleotide to form a stable complex.

In the case of RRM3, no role for binding to U-rich RNA has been reported. It is possible that a low affinity interaction occurs with a non-U-rich sequence. Recently, an NMR based approach was used to screen an RNA pentamer library to find a consensus binding motif for TIA-1 RRM3 [45]. Interestingly, a C-rich motif was discovered and verified by SPR and NMR difference spectroscopy. It may be that RRM3 serves to direct the specificity of binding by the TIA proteins by augmenting binding when this target sequence occurs alongside a U-rich target of RRM2. Indeed, TIAR binding to a C-rich target was enhanced when RRM3 was present, but this same enhancement was not observed for a U-rich RNA target [38]. It has also recently been verified that TIA-1 binds to a UC-rich sequence in the 5'-intron site of pre-FAS mRNA [46]. Thus, it may also be that RRM3 underlies observations of TIAR binding to C-rich RNA in TOP elements [30].

## 5. Structural Insight into RNA Recognition Motifs of TIA/TIAR

Several efforts have been made to elucidate the structures of TIAR proteins and their individual RRM domains, using X-ray crystallographic, NMR spectroscopic and SAXS methodologies. The crystallographic structure of a modified TIA-1 RRM2 domain at 1.95Å adopts a canonical RRM βαββαβ topology with four β-sheets packed against two α-helices [47]. Typically RNA-recognition motif (RRM) domains consist of two conserved consensus motifs referred to as RNP1 (K/R-G-F/Y-G//A-F/Y-V/I/L-X-F/Y) and RNP2 (V/I/L-F/Y-V/I/L-X-N/L). The RNP1 and RNP2 are located in β3 and β1central strands [48]. TIA-1 RRM2 shows high sequence similarity with the *N*-terminal RRM of Poly-A binding protein (PAB) and Sex lethal protein (SXL). Six amino acids engaged in RNA interactions in PAB and SXL are preserved in TIA-1 RRM2 [47]. Chemical shift perturbation analysis of TIA-1 RRM2 binding to 5' UUUUU 3' RNA showed that most of the β-sheet surface was interacting with RNA and RNA recognition modes are similar between TIA-1 and U2AF65 RRMs [44].

**Figure 3:**
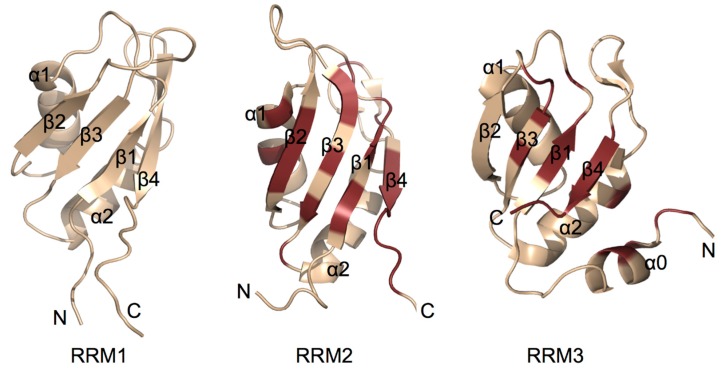
Cartoon representation of TIA protein RNA-recognition motif (RRM) structures solved using NMR spectroscopy. RRM 1 from TIAR (PDB entry:2CQI) and RRMs 2 and 3 from TIA-1 (PDB entry:2MJN) are shown. Amino acid residues of RRMs 2 and 3 shown to be perturbed upon binding by ssRNA are highlighted in ruby showing that binding mainly occurs across the surface the β-sheet, but also involves residues outside the canonical RRM fold [42,44,46,49]. No evidence for specific binding of RNA by RRM1 has been observed.

Structures of the individual TIAR RRMs have been elucidated using NMR by the RIKEN Structural Genomics/Proteomics Initiative. TIAR RRM1 and RRM2 share the typical canonical RRM βαββαβ topology (PDBID: 2CQI and 2DH7). In the case of TIAR RRM3, an additional non-canonical *N*-terminal α0 helix was observed. Subsequently this α0-helix was also shown to be present in TIA-1 and, furthermore, NMR chemical shift perturbation analysis showed that the α0-helix is involved in RNA binding [46]. In both cases of RRM2 and RRM3, binding to RNA was found to predominantly involve amino acid residues across the RNP1 and RNP2 consensus motif regions (Figure 3). In addition, residues *C*-terminal to the structured RRM domain were found to be involved [42,44,46,49]. No high resolution structure of a TIA protein RRM domain in complex with oligonucleotide has yet been reported. Until such structural information is available, the precise structural basis for the RNA binding specificity of TIA proteins will remain unknown.

The low resolution solution shape of TIA-1 RRM123 and its complex with AU rich RNA from the 3' UTR of TNF-α mRNA has been characterized using small angle X ray scattering (SAXS) by Bauer *et al* [41]. Their SAXS analysis revealed that TIA-1 RRM123 exists in an extended arrangement and exists as an obtuse V shape positioning the amino and carboxy termini on the same face of the molecule. However, TIA123 undergoes a conformational change in RNA bound state. These results are consistent with the recent NMR titrations and SAXS studies conducted on a TIA-1 RRM23 construct, which showed that RRM2 and RRM3 tumble independently in solution, but upon binding to RNA derived from FAS pre-mRNA intron 5, the protein-RNA complex becomes rigid and compact [46].

## 6. TIA Protein Self-Association via the Prion Related Domain

Besides the stalling of the translation of mRNA, with which TIA proteins are associated, leading to a general accumulation of TIA/RNA, there is little known about the trigger of SG formation via the prion-related domain (PRD; and also referred to as Q-rich or low complexity domain). Spontaneous self-assembly is the hallmark feature of PRDs, so it is possible that the general accumulation of mRNA associated with TIA proteins could reach a critical concentration upon which SG form via PRD self-association. This would be analogous to the classical model of pathological protein aggregation in neurodegenerative diseases. According to this model, aggregation prone proteins initially exist as monomers. In response to environmental assault, these proteins unfold and can undergo intermolecular association. Heat shock proteins are able to prevent this occurrence to some extent, but when heat shock proteins are unable to prevent the oligomerisation of the misfolded proteins fibrillar aggregates known as amyloid fibrils are eventually produced (for reviews, see refs [50,51]). These fibrils are toxic to the cell causing inflammation and cell death [52,53].

Indeed, in a study of Huntingtin (Htt) protein aggregation, it was shown that full-length TIA-1 forms aggregates spontaneously *in vitro* similarly to the Huntingtin protein [54]. Furthermore, congo red interactions, Thioflavin T fluorescence emission and atomic force microscopy images demonstrated that TIA-1 self-association bears the hallmarks of β-sheet fibril formation. Likewise, Kao *et al.*, have shown that fibril formation by TIA-1 is able to be recapitulated *in vitro* [55]. They observed that a porous hydrogel is produced by TIA-1 and other PRDs that can form quickly once initiated. Analysis of the hydrogel also revealed fibrils that have all of the properties of the classical pathological amyloid except that the structures within are dynamic and able to include several proteinaceous species. This suggests a model of SG organisation in which SG-proteins and RNA are able to diffuse in and become associated with the hydrogel matrix until such time that the hydrogel is disassembled by heat shock proteins.

SG formation is thus similar to the process of pathogenic amyloid formation except for its reversible nature and, indeed, it is now proposed that SG proteins could be involved in such pathogenic processes [56]. Tau inclusions, that are a major hallmark of Alzheimer’s disease, have been shown to co-localize with SGs and it has recently been revealed that TIA-1, directly interacts with the tau protein and induces formation of tau inclusions [57]. Thus, a link between healthy and pathological TIA protein self-association is now emerged. In the case of amyotrophic lateral sclerosis (ALS), the pathogenic TDP-43 protein that is also an RNA binding protein with a prion-like domain, forms inclusions that are co-localised with SG [58]. Accordingly, it may be that strategies that reduce the formation of pathological SGs will have potential against neurodegenerative diseases.

## 7. Conclusions

The dynamic interactions of TIA proteins with target mRNA, other associated proteins and self-association of the PRD underpins a rapid response in gene regulation under conditions of cellular stress. There exists considerable insight into the subset of mRNA that are sequestered into SGs by TIA proteins under such circumstances, and this selectivity has been rationalised at a molecular level to some extent. There has also been progress in the characterisation of the self-associative properties of the TIA proteins that they undergo in the formation of SGs. Further biophysical and cellular investigations are anticipated to explain more precisely the basis of mRNA selection, and also to reveal details of TIA self-association and disassociation mechanisms. Eventually our understanding of this healthy cellular response may allow us to intervene in cases where TIA proteins are associated with a pathological inflammation or aggregate formation.
